# Integration of Artificial Neural Network Regression and Principal Component Analysis for Indoor Visible Light Positioning

**DOI:** 10.3390/s25041049

**Published:** 2025-02-10

**Authors:** Negasa Berhanu Fite, Getachew Mamo Wegari, Heidi Steendam

**Affiliations:** 1 TELIN/IMEC, Ghent University, 9000 Gent, Belgium; heidi.steendam@ugent.be; 2 Faculty of Computing and Informatics, Jimma Institute of Technology, Jimma University, Jimma 378, Ethiopia; getachew.mamo@ju.edu.et

**Keywords:** visible light positioning (VLP), light-emitting diode (LED), received signal strength (RSS), artificial neural network (ANN), principal component analysis (PCA)

## Abstract

The advancement of artificial intelligence has brought visible-light positioning (VLP) to the forefront of indoor positioning research, enabling precise localization without additional infrastructure. However, the complex interplay between light propagation phenomena and environmental factors in indoor spaces presents significant challenges for VLP systems. Additionally, the pose of the light-emitting diodes is prior unknown, adding another layer of complexity to the positioning process. Dynamic indoor environments further complicate matters due to user mobility and obstacles, which can affect system accuracy. In this study, user movement is simulated using a constructed dataset with systematically varied receiver positions, reflecting realistic motion patterns rather than real-time movement. While the experimental setup considers a fixed obstacle scenario, the training and testing datasets incorporate position variations to emulate user displacement. Given these dataset characteristics, it is crucial to employ robust positioning techniques that can handle environmental variations. Conventional methods, such as received signal strength (RSS)-based techniques, face practical implementation hurdles due to fluctuations in transmitted optical power and modeling imperfections. Leveraging machine learning techniques, particularly regression-based artificial neural networks (ANNs), offer a promising alternative. ANNs excel at modeling the intricate relationships within data, making them well-suited for handling the complex dynamics of indoor lighting environments. To address the computational complexities arising from high-dimensional data, this research incorporates principal component analysis (PCA) as a method for reducing dimensionality. PCA eases the computational burden, accelerates training speeds by normalizing the data, and reduces loss rates, thereby enhancing the overall efficacy and feasibility of the proposed VLP framework. Rigorous experimentation and validation demonstrate the potential of employing principal components. Experimental results show significant improvements across multiple evaluation metrics for a constellation comprising eight LEDs mounted in a rectangular structure measuring a room dimension of 12 m × 18 m × 6.8 m, with a photodiode (PD) receiver. Specifically, the mean squared error (MSE) values for the training and testing samples are 0.0062 and 0.0456 cm, respectively. Furthermore, the R-squared values of 99.31% and 94.74% for training and testing, respectively, signify a robust predictive performance of the model with low model loss. These findings underscore the efficacy of the proposed PCA-ANN regression model in optimizing VLP systems and providing reliable indoor positioning services.

## 1. Introduction

The evolution of optical wireless communications (OWC) has revolutionized wireless connectivity across a broad spectrum, from infrared to ultraviolet. Visible light communication (VLC) has emerged as a standout technology, offering immense potential for indoor positioning and high-speed data transmission [1]. With the widespread use of light-emitting diodes (LEDs) for illumination, integrating VLC capabilities into lighting infrastructure has brought about improved efficiency and longevity [2]. VLC positioning techniques include model-based and model-free methods. Model-based techniques, such as time-of-arrival (ToA), time-difference-of-arrival (TDoA), angle-of-arrival (AoA), and received signal strength (RSS) positioning, use mathematical models to estimate distances or angles between transmitters (Tx) and receivers (Rx) [3,4]. These methods face challenges due to mismatches between theoretical models and real-world conditions, such as non-Lambertian radiation patterns, optical power fluctuations, unknown LED poses, reflections, blockages, and shadowing [5,6]. Conversely, model-free techniques, like fingerprinting and proximity-based methods, rely on empirical data [7]. Fingerprinting uses a database of measurements taken at various positions, comparing real-time data to determine location. Although it avoids some model-related issues, it struggles with maintaining an extensive and accurate database in dynamic environments where lighting conditions change due to moving objects or other factors. Proximity-based techniques estimate position by identifying nearby LEDs but generally offer lower accuracy and are similarly affected by environmental changes. Machine learning (ML) techniques extend model-free methods, particularly fingerprinting, to handle dynamic scenarios better and improve data coverage and distribution, enhancing the robustness and accuracy of indoor positioning systems [8]. These advancements in VLC positioning reflect ongoing efforts to refine and optimize positioning technologies for more reliable applications in various environments. To tackle these challenges, there is a growing interest in leveraging machine learning (ML) techniques, notably artificial neural networks (ANNs), which offer enhanced adaptability and resilience in real-world scenarios. Various indoor positioning technologies have begun incorporating ML methods into their localization engines to improve performance [9]. However, the existing approaches often overlook the dynamic indoor environment and the influence of light propagation phenomena and environmental factors on positioning accuracy. In their research, Long et al. propose an indoor visible light positioning system using AI-driven point classification, highlighting the limitations of traditional RSSI-based methods [8]. However, their method does not adequately account for changes in light intensity due to obstacles, reflections, and shadowing, leading to inaccuracies in positioning. Additionally, variations in LED orientation and placement and fluctuations in optical power are insufficiently addressed, compromising the system’s reliability. Long et al. suggest adopting AI algorithms with faster convergence speeds and enhanced capabilities to improve the system. Our research aims to advance VLP systems based on the work by Raes et al. [10]. Raes et al. previously analyzed RSS-based VLP setups, comparing the multi-layer perceptron (MLP) to analytical methods [11]. While their work demonstrated the potential of MLPs in positioning systems, it did not integrate advanced dimensionality reduction techniques like principal component analysis (PCA) into artificial neural networks (ANNs) to reduce the model complexity. By incorporating PCA, we aim to capture significant data variances, thereby improving model complexity, particularly in complex indoor environments. Additionally, Raes et al.’s approach of using multiple less complex models for different regions (cells) may lead to overfitting and higher positioning errors, highlighting the need for a more integrated and robust approach. Dynamic indoor environments pose significant challenges for visible light positioning systems, as user mobility, obstacles, and reflective surface alterations introduce variability into received signals. This study addresses these challenges through the proposed PCA-ANN framework, which combines PCA for dimensionality reduction and ANN for accurate regression. By isolating significant features and filtering noise, the PCA-ANN model mitigates the impact of environmental variability, achieving robust and precise positioning even under complex indoor conditions. The novelty of this study lies in integrating PCA with ANN regression to enhance indoor VLP systems by reducing computational complexity, minimizing overfitting, and improving predictive accuracy. This hybrid approach addresses challenges in high-dimensional data and environmental variability, setting a benchmark for precision and scalability in real-world VLP applications.

The paper follows this structure: Section 2 introduces the system model and communication channel characteristics. Section 3 discusses the neural network architecture and learning algorithm. Section 4 presents simulation results and performance analysis. Finally, Section 5 summarizes our contributions and concludes the work.

## 2. System Model

**Figure 1:**
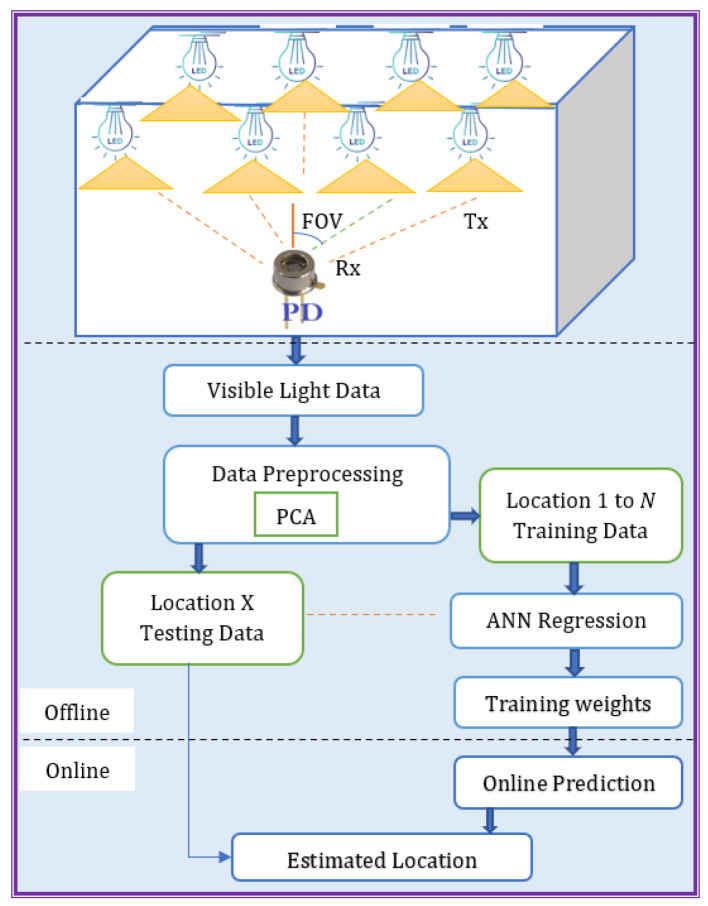
Workflow of the PCA-ANN Regression Model for Indoor VLP.

This paper presents the PCA-ANN regression model for an indoor visible light positioning system using intensity modulation and direct detection. The model aims to predict the numerical output variable, i.e., the position, based on RSS measurements. Figure 1 illustrates the schematic representation of this system. The process begins with multiple LEDs (transmitters, Tx) emitting visible light signals, which are received by a photodiode (receiver, Rx) within its field of view (FOV). This results in RSS measurements that are collected, labeled, and preprocessed for further analysis. To enhance efficiency and accuracy, PCA is applied to reduce the dataset’s dimensionality while preserving essential features. The dataset is then divided into two subsets: (i) Dataset 1 (training data), used to train the ANN model offline and PCA is first applied to Dataset 1 to extract principal components and streamline the dataset, and (ii) Dataset 2 (testing data), used in the online phase, where the PCA transformation derived from Dataset 1 is applied to ensure consistency and prevent data leakage. After preprocessing, the ANN is trained offline by optimizing its weights, enabling real-time predictions during deployment. During the online phase, the trained ANN model processes Dataset 2 and predicts the estimated receiver location based on the learned patterns. This workflow ensures a strict separation between training and testing datasets, guaranteeing reliable and unbiased model evaluation. Ultimately, the estimated position is determined based on insights gained from the analysis.

### 2.1. Communication Model

In this paper, we assume *L* LEDs are positioned at a height *h* above the observation plane of the photodiode receiver (PD). Each LED l=1,…,L emits an intensity-modulated waveform with a distinct fundamental frequency $f_{l}$. This modulation enables the differentiation of light signals based on their respective frequencies. The intensity-modulated waveform emitted by each LED is represented as

(1) $$\begin{array}{c} s_{l}\left(t\right)=\frac{P_{t}}{2}\left[1+\mathrm{sgn}(\sin\left(2\pi f_{l}t+\phi_{l}\right))\right]. \end{array}$$

In this equation, $s_{l}\left(t\right)$ represents the transmitted signal, $P_{t}$ is the optical power transmitted by the LED, $f_{l}$ is the frequency transmitted by LED *l*, and $\phi_{l}$ is a random and unknown phase offset that varies for each LED [12]. The PD receiver r(t) captures the sum of the transmitted signals $s_{l}\left(t\right)$ and converts the optical signals into electrical signals, as given by [13,14]

(2) $$\begin{array}{c} r\left(t\right)=\sum_{l=1}^{L}\alpha_{l}R_{p}s_{l}\left(t\right)+\beta +w\left(t\right), \end{array}$$

where $R_{p}$ is the responsivity of the PD, *L* is the total number of LEDs, β is the DC contribution of ambient light sources, w(t) is the noise component, and $\alpha_{l}$ is the channel attenuation, expressed as

(3) $$\begin{array}{c} \alpha_{l}=f\left(d_{l},A_{r},\theta_{l},\gamma_{l}\right). \end{array}$$

In (3), $d_{l}$ is the Euclidean distance between an LED *l* and the photodetector (PD), $A_{r}$ is the area of the photodetector at the receiver, $\theta_{l}$ is the inclination angle, and $\gamma_{l}$ is the angle of incidence at the PD [5,15].

In the existing literature, $f(d_{l},A_{r},\theta_{l},\gamma_{l})$, which is represented by $u_{l}$, is often modeled mathematically based on a Lambertian radiation pattern [12]. This model assumes that the emitted light intensity is proportional to the cosine of the angle from the normal, simplifying the mathematical representation of the channel. However, real-world LEDs often exhibit intensity patterns that deviate from the idealized Lambertian model because of manufacturing imperfections and design considerations. These deviations are typically characterized using luminous intensity distribution curves (LIDCs), which provide a detailed representation of the emitted light’s angular intensity distribution. For instance, most LEDs display lower intensity at wider angles than the Lambertian model predicts. Such discrepancies can lead to a mismatch between the mathematical model and the true channel characteristics, resulting in performance degradation in optical communication and positioning systems. To avoid such a performance reduction, machine learning techniques offer a compelling alternative. The model learns from data in machine learning rather than relying solely on mathematical formulations. This data-driven approach enables the model to capture complex relationships and adapt to variations in channel characteristics. Considering the multivariate dependency of $u_{l}$ on variables such as the Euclidean distance ($d_{l}$), area ($A_{r}$) of the photodetector, angle of inclination ($\theta_{l}$), and angle of incidence ($\gamma_{l}$), it becomes evident that traditional mathematical models may not be able to capture the intricacies of the channel. This motivates the application of principal component analysis (PCA) in our research. PCA offers a systematic methodology for dimensionality reduction while retaining the key features of the dataset [16]. Given the complex interplay of variables influencing f(n), PCA enables us to distill the essential components of the data, thereby facilitating a more concise representation of the channel characteristics. By leveraging PCA, we aim to address the challenges posed by the channel attenuation function f(n) and contribute to enhanced performance and reliability in optical communication systems.

### 2.2. Principal Component Analysis (PCA)

**Figure 2:**
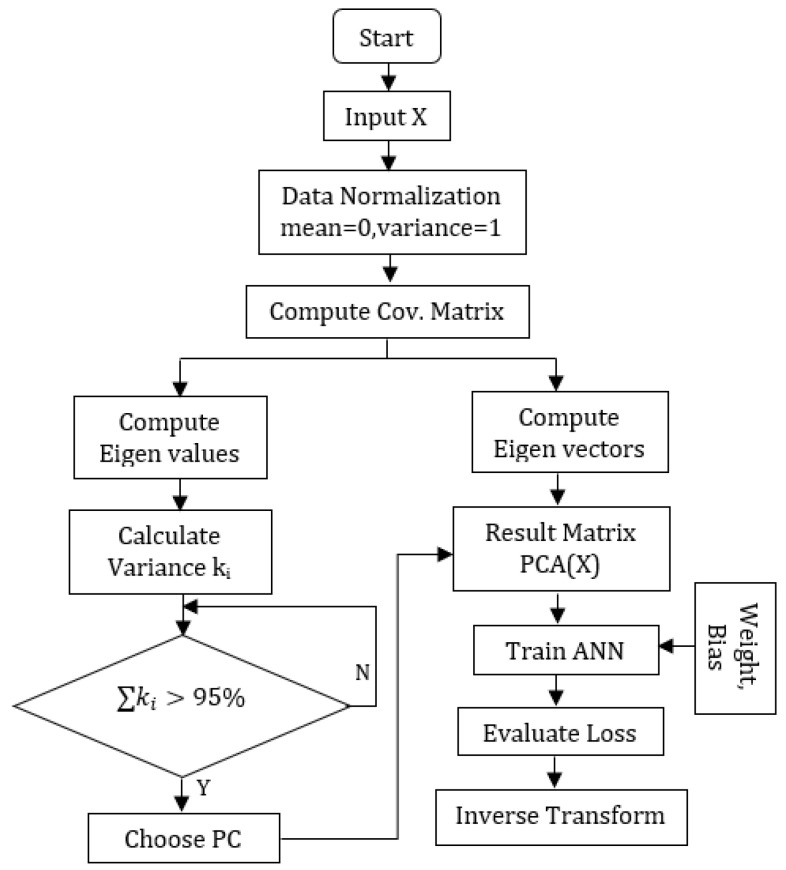
PCA-ANN Flowchart.

PCA enhances ANN learning by reducing dimensionality by simplifying data into orthogonal principal components. However, this reduction in complexity can come at the cost of performance if dominant contributions are removed. To balance model complexity and performance, selecting the optimal number of principal components is crucial. Figure 2 visually illustrates the flowchart for the PCA-ANN regression model, demonstrating the integration of PCA in preprocessing data to improve the efficiency and learning outcomes of the ANN.

In Algorithm 1, *n* observations represent the individual data points or instances in the dataset *X*, each described by a row in the matrix. These observations encapsulate specific measurements or attributes that characterize each data point. Conversely, *p* variables denote the different intensities measured across all observations, typically represented as columns in *X*. These variables collectively define the dataset’s dimensionality and complexity. The algorithm utilizes *X* to preprocess the data by first normalizing it using *mean* and standard deviation *std_dev* vectors calculated along the columns of *X*. Cov(X) represents the covariance matrix of *X*, capturing relationships between variables. Eigenvalues λ and eigenvectors μ of Cov(X) are extracted for principal component analysis (PCA), reducing *X*’s dimensionality while retaining crucial variance information. Cumulative variance is an array or list that contains cumulative variance values calculated from the eigenvalues of the covariance matrix Cov(X). These cumulative variance values indicate the total variance explained by each successive principal component. The chosen threshold, 0.95, aims to retain a significant portion of the variance in the original dataset while reducing its dimensionality. Specifically, *W* is set to the smallest number of principal components needed to retain at least 95% of the total variance, represented as min{k∣cumulative_variance[k]≥0.95}. This notation means finding the smallest index *k* so that the cumulative variance up to the *k*-th principal component is at least 95%. In other words, *W* is set to the principal components needed to retain at least 95% of the original dataset’s variance. The resulting transformed data PCA_X is then fed into an ANN to reduce the complexity. Predictions from the ANN are evaluated using mean squared error (MSE), and inverse-transformed to the original scale using PCA_components, mean, and std_dev.
**Algorithm 1:**PCA-ANN Algorithm**Require:** *X*—Data matrix of size n×p (*n* observations, *p* variables)**Ensure:** Trained ANN with PCA preprocessing1:**Step 1: Normalize data**2:mean←calculate_mean(X,axis=0)3:std_dev←calculate_std_dev(X,axis=0)4:$normalized\_X\leftarrow \frac{X-mean}{std\_dev}$5:**Step 2: Compute Covariance Matrix**6:$Cov\left(X\right)\leftarrow \frac{1}{n-1}\left(normalized\_X^{T}\cdot normalized\_X\right)$7:**Step 3: Compute Eigenvalues and Eigenvectors**8:[λ,μ]←eig(Cov(X))9:**Step 4: Determine the number of Principal Components**10:$cumulative\_variance\leftarrow \mathrm{cumsum}\left(\frac{\lambda }{\sum \lambda }\right)$11:W←min{k∣cumulative_variance[k]≥0.95}12:**Step 5: Perform PCA transformation**13:**Select the top *W* eigenvectors (principal components)**14:PCA_components←μ[:,0:W]15:**Project the normalized data onto the principal components**16:PCA_X←normalized_X·PCA_components17:**Step 6: Train ANN**18:trained_model←train_ANN(PCA_X,labels)19:**Step 7: Evaluate model**20:predictions←trained_model.predict(PCA_X)21:loss←calculate_MSE(predictions,labels)22:**Step 8: Inverse Transform the predicted output (if necessary)**23:predicted_output←inverse_transform(predictions,PCA_components,mean,std_dev)24:**return** trained_model,loss,predicted_output

### 2.3. Artificial Neural Network Regression

**Figure 3:**
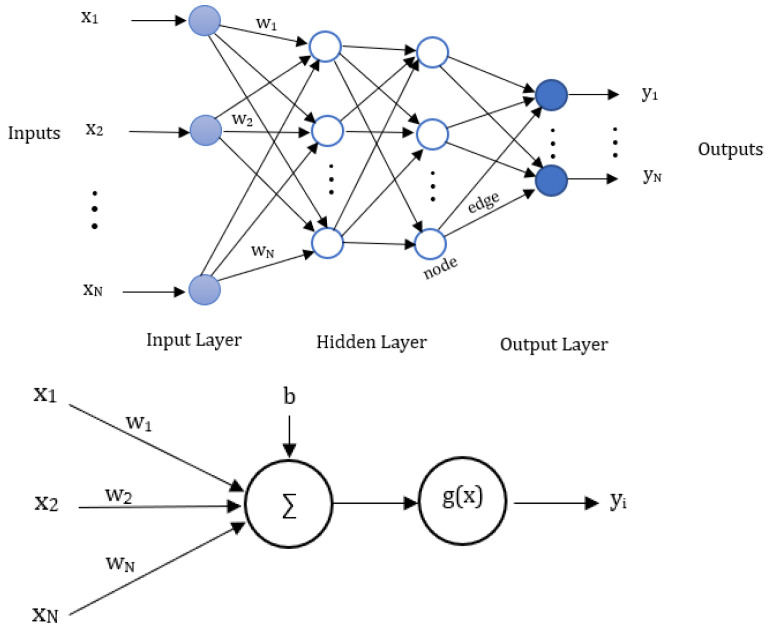
ANN architecture and its mathematical structure.

In our system, we employ the ANN topology depicted in Figure 3, which consists of an input layer, an output layer, and multiple hidden layers. Each node in our network corresponds to a neuron, and matrices can represent the connections between neurons across two layers. We require a topology characterized by robust synaptic connections for precise positioning, ensuring that the combination of connection matrices is not sparse. The functionality of the neural network is determined by the mathematical framework illustrated in Equation (4).

(4) $$y=g\left(\sum_{n=0}^{N}w_{N}x_{N}+b\right)$$

In this context, g(x) represents the activation function, *b* represents the bias, *w* represents the weights that encode the linear relationships in the connection matrices, $X=x_{1},\ldots ,x_{N}$ represent the neuron inputs, and $y=y_{1},\ldots ,y_{N}$ is the output. Each layer of the network intricately relies on its input, as depicted in Figure 3. The activation function plays a crucial role in introducing non-linearity to the network’s dynamics, adjusting the weighted sum from the input layer to the output layer across hidden layers [17]. When a neuron surpasses a specific threshold, it activates and transmits information to the next layer. Initially, all inputs traverse the input layer with random weights. The weighted sum is computed by multiplying each weight by its corresponding input value and summing the results. Introducing a bias *b* into this weighted sum is critical, as the resulting value undergoes the activation function. To dynamically adjust attributes of the neural network, such as weights and learning rate [18], we are going to compare and select slightly better optimization techniques in our results. This iterative process continues as activated neurons are forwarded to subsequent layers, ultimately leading to the output layer where the final prediction is generated.

## 3. Experimental Parameters

**Figure 4:**
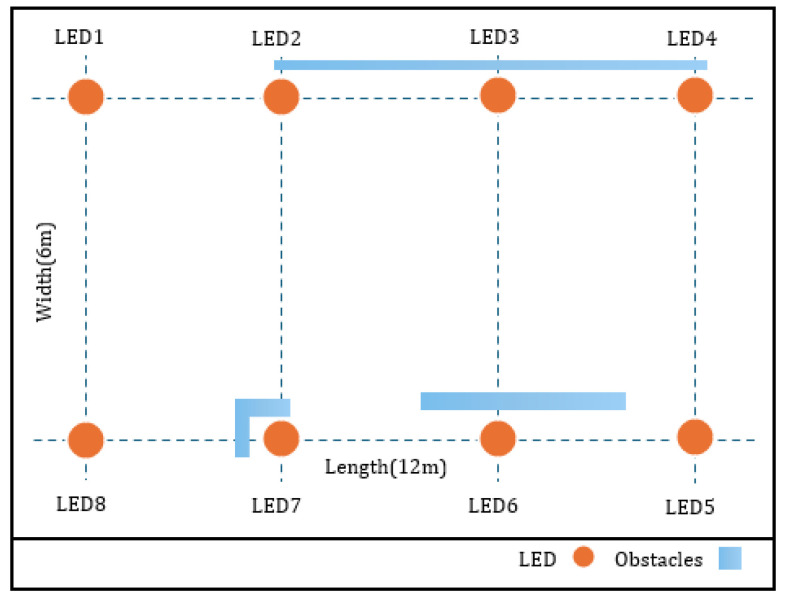
Top view of the Experiment [11].

This section details experiments conducted using datasets from Raes et al. [11]. These datasets encompass measurements taken in a single indoor setting, specifically a hallway environment. Table 1 showcases the experimental system parameters. The experimental setup, illustrated in Figure 4, features eight LEDs arranged in a rectangular formation spanning 6 m by 12 m on the hallway ceiling, alongside various obstacles. To simulate real-world conditions, obstacles in Figure 4 were placed at a height of 1.5 m, designed to partially attenuate rather than completely block light signals. These obstacles introduce variations in received signal strength, which is essential for evaluating positioning accuracy in dynamic environments. The controlled inclusion of obstacles ensures the dataset captures realistic challenges, improving model robustness. For the VLP receiver, a custom photodiode module with a programmable system-on-a-chip (PSOC) was utilized.

### 3.1. Datasets

The dataset utilized to evaluate the PCA-ANN model’s performance consists of 19,360 training samples and 16,771 testing samples, each representing the *x* and *y* coordinates of the receiver’s position. The dataset includes RSS values collected from 8 LEDs, with measurements in an experimental environment designed to simulate real-world conditions. These conditions incorporate dynamic factors such as the movement of people, shifting objects, and changes in obstacles, introducing variability reflective of practical indoor scenarios. To ensure consistency, two distinct datasets were collected in the same experimental environment: Dataset 1, which contains 19,360 samples for training, and Dataset 2, which includes 16,771 samples for testing. These datasets were designed to maintain the original feature distribution across both sets. Before applying PCA, partitioning was conducted to preserve the integrity of the raw feature distribution and ensure compatibility for subsequent model training and evaluation. The PCA-ANN model was trained using the training set, while its performance was continually monitored on a validation set to optimize learning and mitigate overfitting. Once training was completed, the testing set was used to independently evaluate the model’s accuracy in predicting the receiver’s position. This approach ensured a robust assessment of the PCA-ANN model’s ability to leverage RSS values for precise coordinate estimation, even under dynamically varying environmental conditions.

### 3.2. Pearson Correlation Coefficient

To emphasize the significance of PCA before training our dataset with ANNs, we leverage the Pearson correlation coefficient (PCC) heatmap [19]. This visual tool serves as a pivotal preparatory step, highlighting correlations between LED intensities and pose coordinates within our visible light positioning system. By scrutinizing the PCC heatmap, we discern intricate patterns and relationships among variables, which are essential for reducing dimensionality through PCA. Here, *X* represents input variables including timestamps and the intensities of eight lights, while *Y* denotes target variables consisting of pose coordinates (posex, posey, poseyaw). The formula for $r_{X,Y}$, i.e., the PCC between *X* and *Y*, is

(5) $$r_{X,Y}=\frac{\sum_{i=1}^{n}\left(X_{i}-\bar{X}\right)\left(Y_{i}-\bar{Y}\right)}{\sqrt{\sum_{i=1}^{n}{\left(X_{i}-\bar{X}\right)}^{2}}\sqrt{\sum_{i=1}^{n}{\left(Y_{i}-\bar{Y}\right)}^{2}}}$$

where $r_{X,Y}$ quantifies the correlation strength between *X* and *Y*, ranging from +1 to —1. A value of +1 denotes a perfect positive linear correlation, —1 signifies a perfect negative linear correlation, and 0 indicates no linear correlation between the variables. Understanding $r_{X,Y}$ is pivotal as it offers quantitative insights into how variations in LED intensities (represented by *X*) relate to changes in pose coordinates (represented by *Y*). This analysis verifies data reliability and informs subsequent research steps, particularly the enhancement of our visible light positioning system for improved accuracy and performance.

### 3.3. Performance Metrics

We use several metrics to measure accuracy, precision, and explanatory power to evaluate the proposed PCA-ANN regression model’s performance. Among these, we have the mean absolute error (MAE), mean squared error (MSE), and its root, the root mean squared error (RMSE), which assesses precision by measuring the differences between the predicted and actual values [20].

(6) $$\mathrm{MAE}=\frac{1}{m}\sum_{i=1}^{m}\left|a_{i}-p_{i}\right|$$

(7) $$\mathrm{MSE}=\frac{1}{m}\sum_{i=1}^{m}{\left(a_{i}-p_{i}\right)}^{2}$$

(8) $$\mathrm{RMSE}=\sqrt{\mathrm{MSE}}$$

Here, *m* is the number of data patterns in the independent dataset, $a_{i}$ is the actual target for data point *i*, and $p_{i}$ is the prediction of the target for data point *i*. Furthermore, the R-squared ($R^{2}$) value indicates the proportion of variance in the dependent variable explained by the independent variables [21], and the explained variance score (EVS) quantifies the proportion of variance in the target variable explained by the model [22].

(9) $$R^{2}=1-\frac{\sum_{i=1}^{n}{\left(a_{i}-p_{i}\right)}^{2}}{\sum_{i=1}^{n}{\left(a_{i}-\bar{a}\right)}^{2}}$$

(10) $$\mathrm{EVS}=1-\frac{\mathrm{Var}(a-p)}{\mathrm{Var}(a)}$$

Here, $e_{i}$ = $a_{i}$ — $p_{i}$ is the residual error between the actual value $a_{i}$ and predicted value $p_{i}$, Var(a−p) represents the variance of the residual error, and Var(a) denotes the variance of the actual target values. Equations (6)–(10) define these metrics, enabling rigorous evaluation.

## 4. Results and Discussion

In this section, we present the results of our analysis, focusing on the effectiveness of our PCA-ANN-based approach in optimizing the visible light positioning system. We compare dimensionality reduction techniques and explore the impact of various parameters, such as optimizers, which adjust model weights to minimize loss, and learning rates, which control the step size of weight updates to ensure efficient learning. Additionally, we examine the batch size [23], which refers to the number of training samples utilized in one iteration of the training process, and epochs, which determine the number of complete passes through the training dataset. Through optimizing these parameters, we aim to enhance the system’s positioning accuracy and computational efficiency. We evaluate the model’s predictive performance and analyze error metrics, including P50, which provides the median error, and P95, which highlights performance in worst-case scenarios, offering a comprehensive view of model accuracy.

### 4.1. PCA Analysis

**Figure 5:**
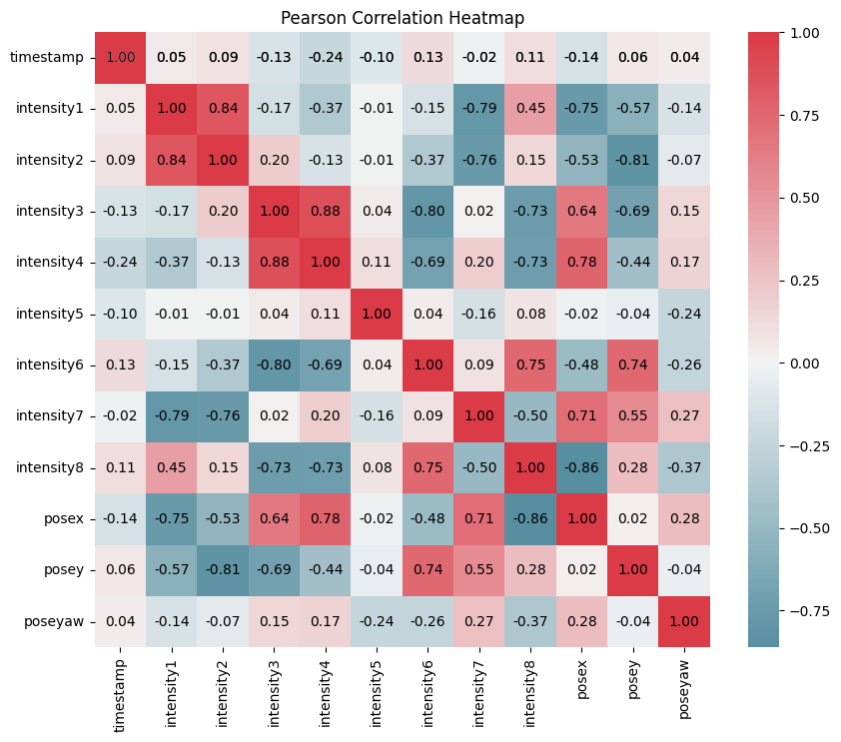
Correlation strength of $r_{X,Y}$ between features.

VLP systems in dynamic indoor environments face challenges due to mobility, obstacles, and environmental variability, which introduce noise and inconsistencies in RSS measurements. To address these issues, experimental measurements in obstacle-rich settings simulated real-world conditions. PCA was then employed to reduce the complexity of high-dimensional data by isolating impactful features and mitigating noise. By preserving essential patterns and discarding irrelevant information, PCA enhanced ANN training efficiency and improved the system’s robustness to environmental changes, ensuring more reliable and accurate positioning performance in dynamic environments. Referring to the Pearson correlation coefficient section, we visualized the heatmap depicted in Figure 5, encompassing nine features: the timestamp and eight intensity measurements (intensity 1 to intensity 8) and three positional measurements (posex, posey, poseyaw). The diagonal elements exhibit a perfect correlation of 1, indicating that each feature is perfectly correlated with itself. Notably, there are strong positive correlations between certain intensity measurements, such as intensity 3 and intensity 4 (0.88) and intensity 6 and intensity 8 (0.75). These strong correlations imply that these intensity measurements capture similar phenomena or are influenced by the same factors. For instance, the intensity of LED 1 shows a positive correlation with LED 2 (0.84) and LED 8 (0.45), reflecting their close spatial proximity and collaborative illumination effects. Conversely, LED 1 exhibits a negative correlation with LED 7 (−0.79) due to obstructed paths, indicating physical obstacles that hinder direct illumination. These insights, visually represented in the Pearson correlation heatmap in Figure 5 and further illustrated from a top view in Figure 4, lay the foundation for our subsequent PCA. The heatmap effectively reveals the complex interrelationships between features, identifying clusters of strongly correlated variables and significant positive or negative correlations, which are essential for applying PCA before training an ANN.

Building on the findings from Figure 5, this study employed PCA for dimensionality reduction. Table 2 presents the results of a PCA conducted on a dataset, summarizing the explained variance by each principal component (PC), their eigenvalues, and both the individual and cumulative variance contribution rates (CR and Cumulative CR). The first principal component (PC1) accounts for 35.3% of the total variance with an eigenvalue of 2.757. Adding the second principal component (PC2), which explains an additional 32.8% variance with an eigenvalue of 2.558, the cumulative variance reaches 68.1%. Including the third component (PC3) brings the total to 85.4% with an eigenvalue of 1.350. The fourth component (PC4) adds 11.1% variance, increasing the cumulative total to 96.4%, which surpasses the 95% cutoff threshold we set for our analysis. This indicates that the first four principal components are sufficient to capture over 95% of the total variance in the dataset, making them a suitable choice for dimensionality reduction. This selection helps to simplify the dataset while preserving most of the original information, facilitating more efficient and effective modeling.

**Figure 6:**
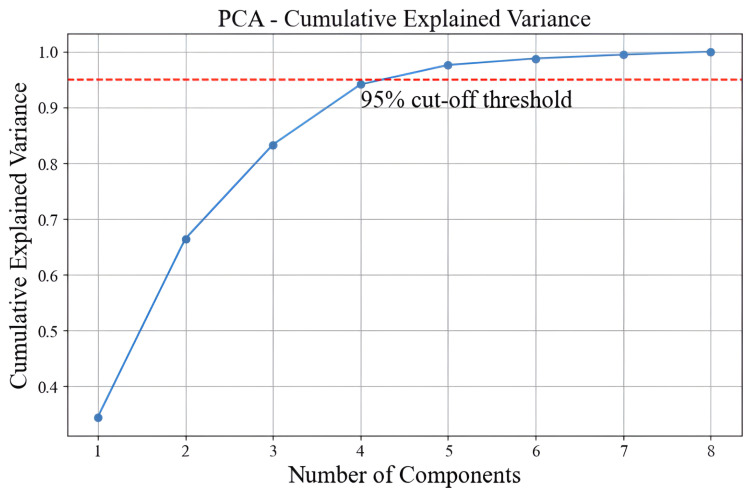
Cumulative explained variance ratio for the number of dimensions (PCA).

**Figure 7:**
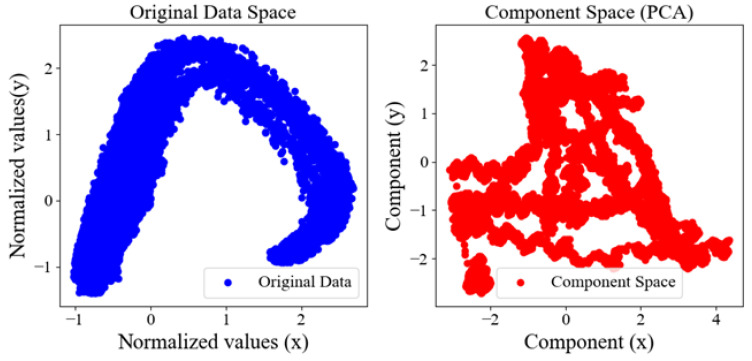
Original vs. Component Space.

Table 2 presents the explained variance ratio for the first four principal components (PCs), which meet the 95% cut-off threshold. A similar result is obtained for the total variance, which is shown in Figure 6. Furthermore, here, four principal components are required to have a total variance of over 90%. In other words, we can reduce the dimensionality of the dataset from eight RSS measurements to four principal components while retaining most of its variance. This simplification enhances the performance of ANN by focusing on the most informative data aspects. Figure 7 compares data in its original form with its transformed state after applying PCA. In the original data space plot, blue dots represent observations based on two normalized features, forming a distinct arch-like shape indicative of a non-linear relationship. This plot highlights the complexity and structure of the raw data. In the component space (PCA) plot, red dots represent the same observations transformed into a lower-dimensional space defined by *x* and *y* components. This transformation simplifies the data, capturing the most significant variance while retaining the underlying structure. The comparison illustrates PCA’s role in simplifying complex data while preserving critical information.

### 4.2. Comparison of Dimensionality Reduction Techniques

**Figure 8:**
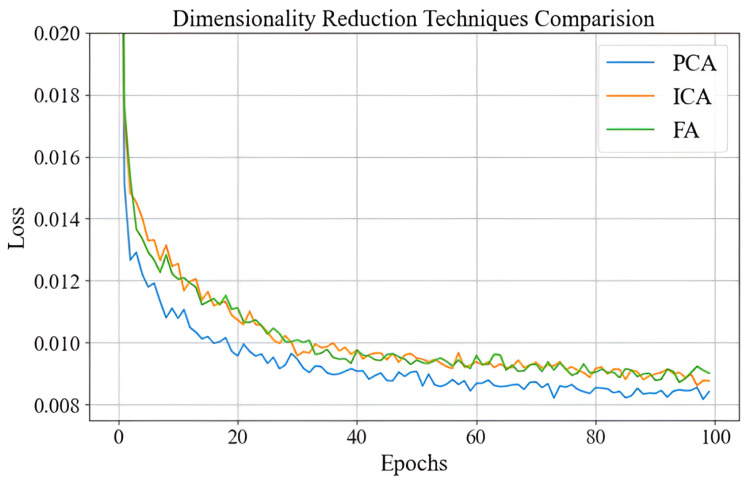
Dimensionality Reduction Technique Comparison.

Independent component analysis (ICA) and factor analysis (FA) are linear dimensionality reduction techniques commonly applied in regression tasks, each with distinct methodologies [24]. ICA focuses on decomposing a dataset into statistically independent components, making it particularly useful for applications such as signal separation. In contrast, FA models observed variables as linear combinations of underlying latent factors, emphasizing the covariance structure of the data. While both techniques are effective in specific domains, they are less efficient than principal component analysis (PCA) in terms of variance capture and computational performance. As illustrated in Figure 8, PCA consistently outperforms ICA and FA in reducing loss over 100 epochs when applied as a dimensionality reduction method for a neural network model. PCA begins with a lower initial loss of approximately 0.018 and quickly decreases to about 0.009 within the first 20 epochs. In comparison, ICA and FA start with slightly higher initial losses of approximately 0.019 and reduce more gradually, reaching 0.010 and 0.011, respectively, by the 20th epoch. Over the full training period, PCA achieves a final loss of approximately 0.0085, outperforming ICA (0.0095) and FA (0.0098).

This performance difference demonstrates that PCA achieves about a 10% lower final loss compared to ICA and an approximately 13% lower final loss than FA. The faster convergence and lower overall loss values underscore PCA’s superior ability to capture variance, enabling it to extract more informative features for the neural network model. Combined with its computational efficiency, PCA emerges as the preferred choice for dimensionality reduction in this context, outperforming ICA and FA by significant margins throughout the training process.

### 4.3. Effectiveness of PCA-ANN with Learning Rate

**Figure 9:**
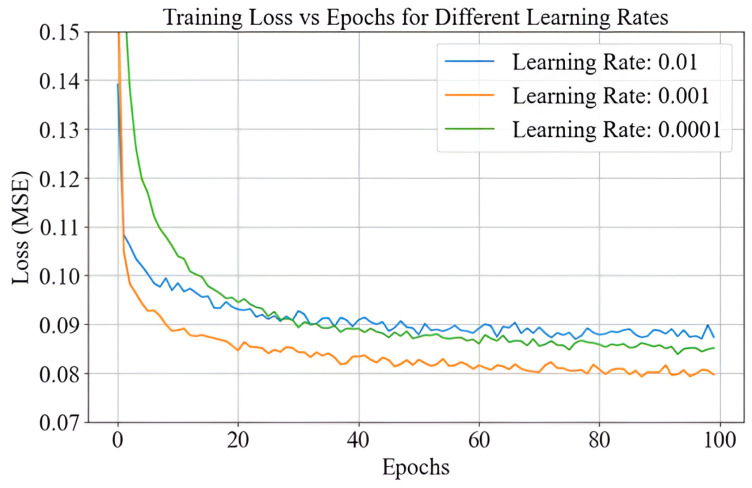
Learning rate comparison.

The learning rate is a hyperparameter in neural network training that determines the step size for weight updates during optimization. It is essential to balance convergence speed and stability, ensuring effective learning without overshooting or stagnating [25]. A high learning rate can cause instability, resulting in fluctuations or divergence, while a low learning rate slows convergence or traps the model in suboptimal solutions [18]. Figure 9 shows the training loss over 100 epochs for a neural network trained with three learning rates (0.0001, 0.001, and 0.01). Initially, all learning rates show a rapid decrease in training loss, indicating effective initial learning. However, their behaviors diverge as training progresses. A learning rate of 0.001 achieves the best balance between speed and stability, stabilizing at the lowest training loss. This is because the moderate step size allows the optimizer to make consistent progress toward the minima, navigating both sharp and flat regions of the loss surface effectively. In contrast, a learning rate of 0.0001 results in smaller weight updates, leading to slower progress and convergence at a higher loss value, as the model struggles in flat regions or shallow gradients. Meanwhile, a learning rate of 0.01 causes large weight updates that overshoot the minima, resulting in significant fluctuations and difficulty in achieving convergence. These observations highlight the importance of selecting an appropriate learning rate in the PCA-ANN framework. A moderate learning rate of 0.001 ensures effective optimization by balancing step size and stability, enabling smooth convergence. In contrast, excessively high or low learning rates hinder the optimizer’s ability to navigate the loss surface efficiently, reducing the model’s performance.

### 4.4. Optimizer Selection

**Figure 10:**
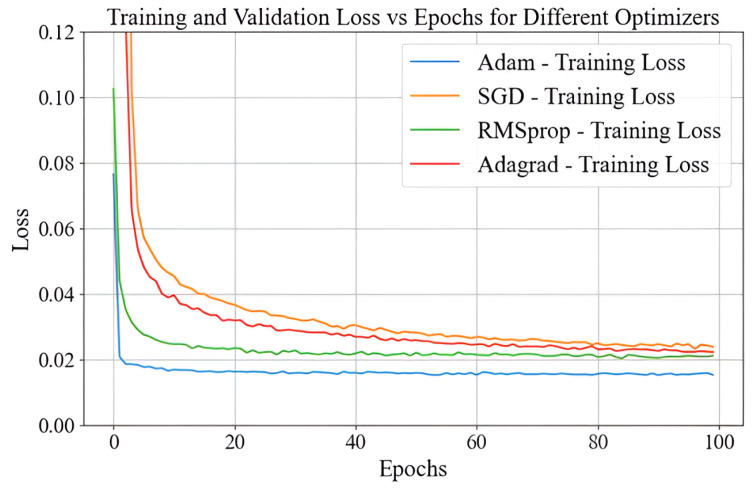
Optimizer Comparison.

In this section, we compare the performance of various optimization algorithms, including adaptive moment estimation(Adam), root mean square propagation (RMSProp), Adagrad, and stochastic gradient descent (SGD), to determine which optimizer facilitates quicker and more effective convergence during training. Optimizers are a crucial part of the model training process, as they adjust the model parameters to minimize the loss function, ultimately improving the model’s ability to learn from data and generalize to new, unseen data. The optimization algorithm is applied during the training phase, after initializing the model and setting up the architecture (in this case, the PCA-ANN model). It operates in each iteration to adjust the weights and biases of the network in response to the gradients calculated from the loss function, effectively driving the model toward the optimal solution. Adam is particularly effective due to its adaptive learning rate and momentum, which help it navigate the loss surface more efficiently [18]. RMSProp adapts the learning rate based on the moving average of recent gradient magnitudes, making it effective for handling non-stationary objectives. Adagrad, which adjusts the learning rate based on gradient frequency, works well for sparse data by making larger updates for infrequent parameters and smaller ones for frequent parameters. SGD, the most basic optimization algorithm, uses the gradient of the loss function to update parameters based on small mini-batches. Figure 10 illustrates the performance of these optimizers with the MSE loss function across 100 epochs at a learning rate of 0.001. The results indicate that Adam outperforms the other optimizers by achieving significantly lower MSE values, particularly in the early stages of training. This is because Adam combines momentum, which smooths the updates by incorporating past gradients, with adaptive learning rates, allowing it to handle large and noisy gradients effectively. In the initial epochs, when gradients are more volatile, Adam’s ability to adjust the step size dynamically ensures faster convergence compared to other optimizers, such as SGD and Adagrad, which rely on fixed or decaying learning rates. By maintaining stability in high-gradient regions and avoiding oscillations in flat areas of the loss surface, Adam achieves both speed and precision in optimization. This observation is consistent with Figure 10, where Adam’s curve demonstrates the steepest initial drop in loss, stabilizing at the lowest final value. In contrast, SGD exhibits the slowest convergence, maintaining relatively high loss values throughout training. RMSProp and Adagrad show moderate performance, converging faster than SGD but failing to match Adam’s efficiency. Although all optimizers eventually converge to similar MSE values, Adam’s faster reduction in MSE highlights its efficiency in minimizing the loss function. As a result, Adam was selected for use in the PCA-ANN model to ensure more efficient and effective optimization, ultimately enhancing model performance and reducing training time.

### 4.5. Batch Size Impact

**Figure 11:**
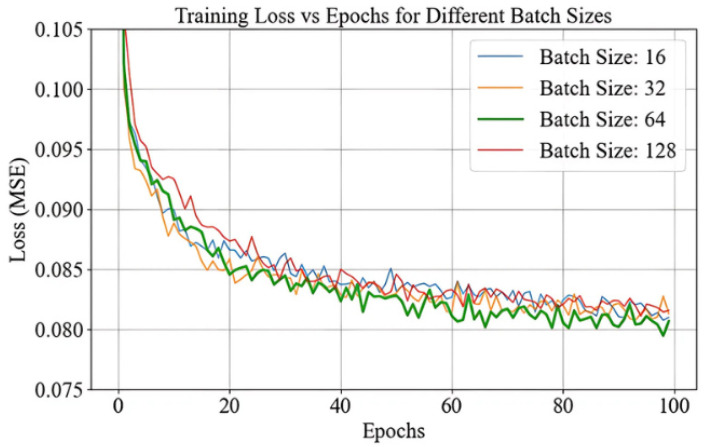
Batch Size Performance.

The plot in Figure 11, illustrates the training loss (MSE) across epochs for different batch sizes (16, 32, 64, and 128), highlighting how batch size influences the convergence dynamics and the final performance. As the batch size increases, the loss curves become smoother, with reduced variability and more stable convergence [26]. However, larger batch sizes require more memory and computational resources, which can pose challenges in constrained environments. Conversely, smaller batch sizes introduce greater variability in gradient updates, aiding exploration of the loss surface and helping to find flatter minima that generalize better [23]. However, this increased variability can lead to slower and less consistent convergence due to noisier optimization. For our PCA-ANN model, a batch size of 64 was selected as it strikes an effective balance between training stability and efficient convergence. The plot shows that it provides smooth and steady convergence with minimal fluctuations compared to smaller batch sizes such as 16 and 32, ensuring stable and reliable gradient updates. It also avoids the poor generalization typically associated with large batch sizes, like 128, which tend to converge to sharp minima. Additionally, a batch size of 64 optimizes computational resource usage, making it suitable for our system constraints. This choice enables the model to achieve robust training performance and effective generalization while maintaining convergence stability and computational feasibility.

### 4.6. Selection of Optimal Dropout Rate

**Figure 12:**
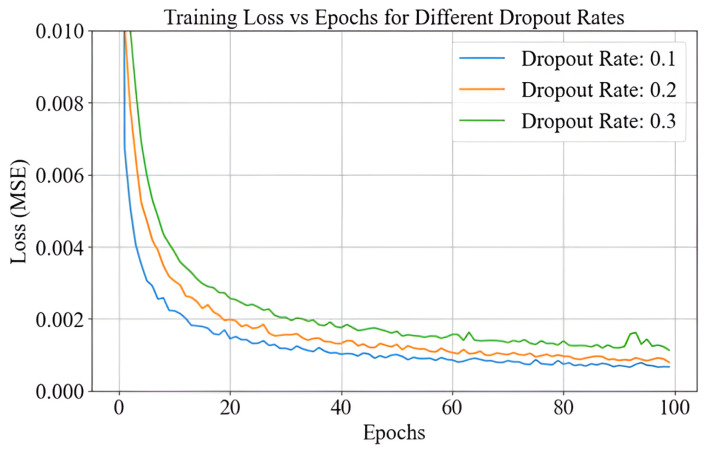
Optimal dropout rate selection.

Dropout is a regularization technique used in neural networks to prevent overfitting by randomly deactivating a proportion of neurons during training. The dropout rate refers to the fraction of neurons that are randomly set to zero during each training step. A higher dropout rate deactivates more neurons, increasing the regularization effect, while a lower rate retains more information in the network [27]. Based on the analysis of the dropout rates shown in Figure 12, a dropout rate of 0.1 was selected as the optimal choice for our PCA-ANN model. This decision was made because, among the tested rates (0.1, 0.2, and 0.3), the 0.1 rate consistently resulted in the lowest training loss over 100 epochs. It allowed the model to generalize effectively without excessively discarding valuable information. As the dropout rate increased to 0.2 and 0.3, stronger regularization was introduced, but this also led to higher training losses, particularly in the early epochs. This behavior suggests that higher dropout rates hindered the model’s ability to capture important patterns in the data. Since PCA already reduces dimensionality and minimizes noise, applying high dropout rates on top of this was detrimental. These higher dropout rates restricted the amount of information passed through the network, making it harder for the model to learn important features. Thus, a dropout rate of 0.1 provided the best balance between regularization and learning stability, promoting effective generalization while allowing the network to retain its ability to capture meaningful patterns in the data.

### 4.7. Hyperparameter Optimization

An extensive hyperparameter tuning process was performed to ensure optimal performance of the PCA-ANN model. Various combinations of learning rate, batch size, and dropout rate were tested to determine their impact on model accuracy and convergence speed. The results of different hyperparameter configurations are summarized in Table 3. The results indicate that a learning rate of 0.001, batch size of 64, and dropout rate of 0.1 achieved the lowest MSE of 0.005cm and the highest R-squared of 99.31%, making this configuration the most optimal for training. Furthermore, increasing the dropout rate to 0.2 resulted in a slight decrease in performance, as seen in the increased MSE of 0.008 cm. Notably, when the learning rate was increased to 0.01, model performance significantly degraded, with an MSE of 0.095 cm. Similarly, reducing the batch size to 32 generally led to an increase in MSE and a slight reduction in the accuracy of the model, reinforcing the importance of selecting an appropriate batch size. These comparisons highlight the importance of hyperparameter tuning in maximizing model efficiency and accuracy, with a careful balance between learning rate, dropout rate, and batch size to prevent overfitting and underfitting.

### 4.8. Model Evaluation

Table 4 presents the performance metrics for the ANN and PCA-ANN models evaluated on training and testing datasets, as detailed in the performance metrics section. In the training phase, the PCA-ANN model performs better with an R-squared value of 99.31% compared to 97.14% for the ANN model. This indicates that our model explains a higher proportion of the variance in the training data. Additionally, the model exhibits lower error values across various metrics with an MSE of 0.0062 cm, MAE of 0.0532 cm, and RMSE of 0.0787 cm. In contrast, the ANN model shows an MSE of 0.0292 cm, MAE of 0.1124 cm, and RMSE of 0.2225 cm. In the testing phase, the PCA-ANN model continues to outperform the ANN model, achieving an R-squared value of 94.74% compared to 91.04% for the ANN model. The error metrics also favor the PCA-ANN model, which has an MSE of 0.0456 cm, MAE of 0.1456 cm, and RMSE of 0.1890 cm. In comparison, the ANN model records an MSE of 0.0989 cm, MAE of 0.1567 cm, and RMSE of 0.2850 cm.

**Figure 13:**
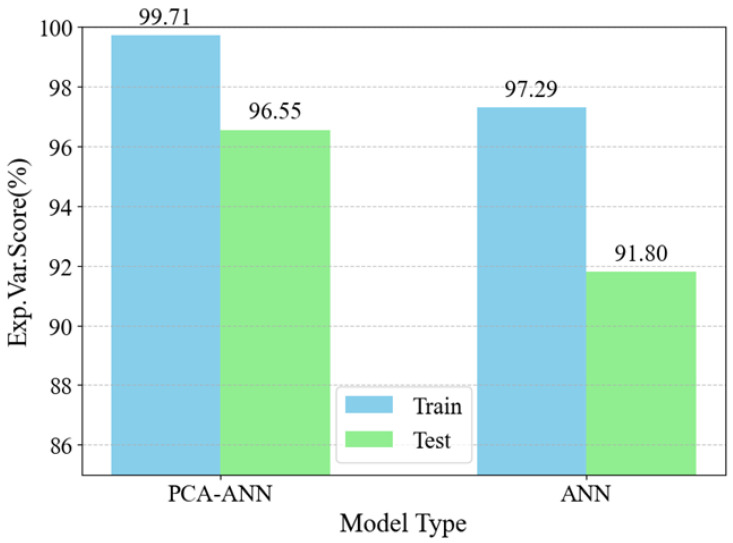
Explained Variance Score Comparison.

Figure 13 shows the explained variance score (EVS) for the training and testing phases of both models, highlighting the PCA-ANN model’s better predictive performance. EVS measures how well the model explains the variability in the data, with higher values indicating better model accuracy. The PCA-ANN model achieves an EVS of 99.71% for training and 96.55% for testing, demonstrating strong data pattern recognition and predictive accuracy. In comparison, the ANN model has slightly lower EVS values of 97.29% for training and 91.80% for testing. These results confirm the PCA-ANN model’s enhanced accuracy and reliability over the standard ANN model in both phases.

### 4.9. Comparison of Actual and Predicted Values

**Figure 14:**
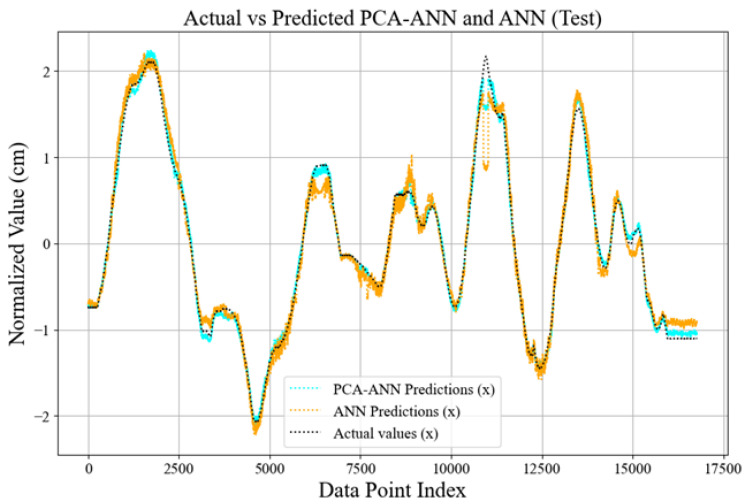
Comparison of actual vs. predicted *x*-coordinates for the test dataset.

**Figure 15:**
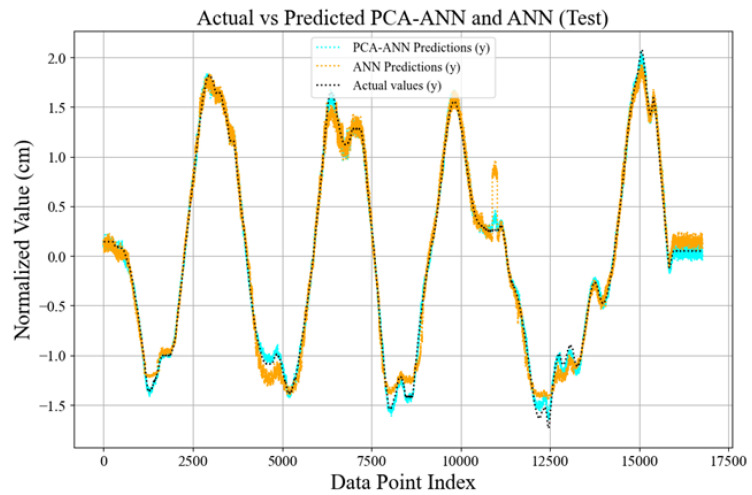
Comparison of actual vs. predicted *y*-coordinates for the test dataset.

The comparative plots in Figure 14 and Figure 15 illustrate the performance of the PCA-ANN model versus the traditional ANN model in predicting the test data set values. The predictions of both models for the coordinates *x* and *y* are plotted alongside the normalized actual values, which represent the ground truth values derived from the test dataset and are scaled during pre-processing. In Figure 14, the actual values represent the ground truth *x* coordinates obtained from the test dataset, which has been normalized during pre-processing to ensure consistent scaling and compatibility with the model’s training framework. These values are used as the benchmark for assessing the accuracy of the model’s predictions. The predicted values are the output generated by the PCA-ANN and ANN models, reflecting the estimated *x* coordinates based on the learned patterns from the input data. The plot demonstrates that the PCA-ANN model aligns more closely with the actual values compared to the ANN model, especially in regions with rapid fluctuations, indicating its improved stability and accuracy in predicting test data. In both figures, the PCA-ANN model consistently demonstrates smoother convergence toward the actual values with fewer fluctuations than the ANN model. This indicates that PCA-ANN captures patterns with greater stability, likely due to the dimensionality reduction provided by PCA, which enhances generalization and reduces overfitting. For the *y*-coordinates (Figure 15), the PCA-ANN predictions closely align with the actual values across most data points, showing improved accuracy and reduced noise relative to the ANN model. Similarly, for the *x*-coordinates (Figure 14), the PCA-ANN predictions exhibit a closer match to the actual values, particularly in regions with rapid fluctuations. These results validate the effectiveness of our model in enhancing predictive performance by balancing accuracy and computational efficiency.

### 4.10. Comparison of PCA-ANN and MLP Cellular Model

The comparative analysis of the PCA-ANN and MLP Cellular models, proposed by Raes et al. [11], is presented in Table 5 and Table 6. The comparison highlights that while both models share similar architecture (inputs, layers, and node configuration) and rely on RSS for positioning, the PCA-ANN model significantly outperforms the MLP Cellular model regarding precision and scalability. By incorporating the principal component for dimensionality reduction, the PCA-ANN model reduces complexity, prevents overfitting, and enhances feature extraction. This results in a median error (P50) of 0.49 cm and a P95 error of 1.36 cm, compared to 4.3 cm and 16.6 cm, respectively, in the MLP Cellular model. In addition, the PCA-ANN model utilizes a unified dataset and advanced optimization techniques, such as the Adam optimizer with a learning rate of 0.001, ensuring faster convergence and stability during training. In contrast, the MLP Cellular model requires separate classifiers for each subspace, adding complexity and reducing scalability. Moreover, the higher parameter count of the PCA-ANN model (19,360 versus approximately 3218 in the MLP Cellular model) enables it to capture intricate relationships more effectively, improving its predictive performance. Overall, the PCA-ANN approach represents a more robust and scalable solution for visible light positioning, addressing the limitations inherent in the cellular model.

## 5. Conclusions

This study introduces a robust and scalable framework for indoor VLP systems by integrating PCA with neural networks. The proposed model addresses critical challenges in high-dimensional data processing and dynamic indoor environments, achieving higher performance compared to traditional approaches. By leveraging PCA for dimensionality reduction, the model preserves essential data features while significantly improving computational efficiency and reducing overfitting risks. Experimental results demonstrate the model’s remarkable accuracy, with R-squared values of 99.31% and 94.74% for training and testing, respectively. Furthermore, the model achieves an MSE as low as 0.0062 cm in training and 0.0456 cm in testing, along with substantial reductions in median (P50) and worst-case (P95) errors compared to the MLP Cellular model. These findings confirm the robustness and scalability of the PCA-ANN framework, which outperforms the MLP cellular model in precision, error minimization, and operational simplicity. In addition to performance gains, this study also provides a comprehensive analysis of hyperparameter optimization. Through detailed tuning, the best configurations for the learning rate, batch size, dropout rate, and optimization selection were identified. The findings confirm that the Adam optimizer, with a learning rate of 0.001 and a dropout rate of 0.1, strikes the optimal balance of stability, efficiency, and accuracy for training the model. This optimization ensures that the model is not only precise but also computationally efficient, contributing to its scalability and adaptability in real-world applications. The proposed PCA-ANN framework sets a new benchmark for precision and reliability in indoor VLP systems. It offers robust predictive capabilities and seamless adaptability to varying conditions. This work underscores the transformative potential of combining dimensionality reduction techniques with machine learning to enhance the efficiency and scalability of positioning systems. The promising results of this study open new avenues for further research. Future work should explore extending the framework to support three-dimensional positioning and incorporating advanced filtering methods like Kalman filtering for real-time tracking. Additionally, investigating hybrid deep learning architectures that incorporate recurrent or transformer-based networks could further enhance adaptability to dynamic environments. Integrating multimodal sensor data may also significantly improve localization accuracy and system robustness. By establishing a scalable and precise VLP framework, this study provides a critical foundation for future advancements in the field, facilitating the broader adoption of visible light communication and positioning technologies in smart environments.

## Figures and Tables

**Table 1:** Experimental System Parameters [11].

System Parameters	Parameter Value
**Simulation Space**	
Room dimensions	12 m × 18 m
Room height	6.81 m
Receiver placement height	1.1 m
Distance between Tx and Rx	5.71 m
Dataset taken	5 cm inter-distance
**Optical Transmitter**	
Number of LEDs	8
Dimension of LED grid	6 m × 12 m
LED power	25 W
LED bandwidth	3 MHz
Data rate	2 Mbps
**Optical Receiver**	
Photodiode area size	13 mm^2^
Transimpedance gain	40 k
DC-bias voltage	1.024 V
Low pass filter cut-off frequency	36 kHz
Sampling frequency	128 kHz
ADC range	2.048 V
ADC resolution	14-bit

**Table 2:** Explained Variance, Eigenvalues, and Contribution Rates.

PC	Explained Variance	Eigenvalues	CR (%)	Cumulative CR (%)
PC1	0.345	2.757	35.314	35.314
PC2	0.320	2.558	32.762	68.076
PC3	0.169	1.350	17.294	85.370
PC4	0.108	0.864	11.060	96.430
PC5	0.035	0.279	3.570	100.000

**Table 3:** Impact of Hyperparameter Combinations on the PCA-ANN Model Performance.

Learning Rate	Dropout Rate	Batch Size	Mean Test Score	MSE	R² Score
0.001	0.1	64	0.016	0.006	0.993
0.001	0.2	64	0.021	0.008	0.988
0.001	0.1	32	0.020	0.010	0.984
0.010	0.1	64	0.057	0.095	0.882
0.010	0.1	32	0.045	0.013	0.980
0.010	0.2	64	0.048	0.018	0.963
0.0001	0.1	64	0.031	0.010	0.986
0.0001	0.1	32	0.028	0.010	0.986

**Table 4:** Comparison of PCA-ANN and ANN Models.

Metrics	PCA-ANN Train	ANN Train	PCA-ANN Test	ANN Test
R-squared (%)	99.31	97.14	94.74	91.04
MSE (cm)	0.0062	0.0292	0.0456	0.0989
MAE (cm)	0.0532	0.1124	0.1456	0.1567
RMSE (cm)	0.0787	0.2225	0.1890	0.2850

**Table 5:** Similarities Between PCA-ANN and MLP Cellular Models.

Model Property	PCA-ANN Model	MLP Cellular Model
Number of Inputs	8	8
Number of Hidden Layers	3	3
Nodes per Layer	64-32-16	64-32-16
Number of Hidden Nodes	112	112
Learning Type	Supervised	Supervised
Error Metric	Euclidean Distance	Euclidean Distance
Environment Size	12 m × 18 m	12 m × 18 m
Feature Source	LED Signal Intensities	LED Signal Intensities
LED Configuration	Rectangular Grid (8 LEDs)	Rectangular Grid (8 LEDs)
Receiver Device	PD based	PD based

**Table 6:** Comparison of PCA-ANN and MLP Cellular Models.

Aspect	PCA-ANN Model	MLP Cellular Model	Key Advantage of PCA-ANN
Dimensionality Reduction	PCA applied	None	Simplifies data, reducing complexity and improving feature selection.
P50 Error (cm)	0.49	4.3	Reduces the median error by 88.3%, showing significant precision.
P95 Error (cm)	1.36	16.6	Reduces the worst-case error by 91.8%, improving robustness.
Trainable Parameters	19,360	3218	Captures intricate patterns, improving accuracy.
Optimizer	Adam (LR: 0.001)	Not specified	Ensures faster and stable convergence.
Dataset	Unified dataset	Divided by cells	Simplifies training by eliminating the need for separate datasets.
Classification	None	KNN (98.7%)	Removes dependency on subspace classifiers.
Scalability	High	Moderate	Scales better to larger or more complex setups with fewer adjustments.
Complexity	Simple	High	Avoids the need for cell-specific models and classifiers.
Overfitting Risk	Low (due to PCA)	Moderate	Reduces overfitting by emphasizing essential features.
Optimization Method	PCA-enhanced ANN	Direct MLP	Balances complexity and accuracy through preprocessing.

## Data Availability

The datasets used and/or analyzed in this paper can be obtained from the corresponding author upon reasonable request.
